# Taxonomically Restricted Genes Are Fundamental to Biology and Evolution

**DOI:** 10.3389/fgene.2018.00407

**Published:** 2018-09-20

**Authors:** Brian R. Johnson

**Affiliations:** Department of Entomology and Nematology, Center for Population Biology, University of California, Davis, Davis, CA, United States

**Keywords:** taxonomically restricted genes, novel genes, toolkit genes, lineage specific traits, lineage specific genes, evolution of novelty

## Abstract

Genes limited to particular clades, taxonomically restricted genes (TRGs), are common in all sequenced genomes. TRGs have recently become associated with the evolution of novelty, as numerous studies across the tree of life have now linked expression of TRGs with novel phenotypes. However, TRGs that underlie ancient lineage specific traits have been largely omitted from discussions of the general importance of TRGs. Here it is argued that when all TRGs are considered, it is apparent that TRGs are fundamental to biology and evolution and likely play many complementary roles to the better understood toolkit genes. Genes underlying photosynthesis and skeletons, for example, are examples of commonplace fundamental TRGs. Essentially, although basic cell biology has a highly conserved genetic basis across the tree of life, most major clades also have lineage specific traits central to their biology and these traits are often based on TRGs. In short, toolkit genes underlie what is conserved across organisms, while TRGs define in many cases what is unique. An appreciation of the importance of TRGs will improve our understanding of evolution by triggering the study of neglected topics in which TRGs are of paramount importance.

## Introduction

Taxonomically restricted, or lineage specific, genes are genes that are found only in a particular clade or species (Wilson et al., 2005; Begun et al., 2007; Khalturin et al., 2009). An orphan gene, for example, is a gene found in only one species, while an arthropod-specific gene is a gene found throughout the phylum Arthropoda, but in no other clades. The last 10 years have seen increasing interest in the study of TRGs for several reasons. First, genomic sequencing studies have shown that 10–20% of genes in a given species do not have homologs in other species (reviewed in Khalturin et al., 2009). Second, an increasing number of experimental studies have shown that TRGs are important for phenotypic novelty (Toll-Riera et al., 2009; Johnson and Tsutsui, 2011; Tautz and Domazet-Loso, 2011; Ding et al., 2012; Ranz and Parsch, 2012; Li et al., 2013; Babonis et al., 2016). These studies have been conducted on species across the tree of life and for many traits (Wilson et al., 2005; Voolstra et al., 2011; Franzenburg et al., 2013; Li et al., 2013; Shigenobu and Stern, 2013; von Reumont et al., 2014; Zhao et al., 2015). A consensus from this work might be that although a toolkit of conserved genes is central to biology, many cases of evolutionary novelty are associated with TRGs. In the present review, it is argued that this consensus is too conservative and that TRGs are fundamental to biology in general.

We begin with a brief review of the some of the work that has been conducted to date on TRGs. We then introduce the main thesis of the paper, which is that although every organism shares a conserved toolkit of genes, each also has an equally fundamental, and large set of lineage specific genes that underlie the many lineage specific traits that are central to their biology. To give the best example, plants photosynthesize, but animals do not. Chloroplasts rely on the use of many TRGs limited in distribution to plants and other photosynthesizing organisms. Such genes are not part of a conserved toolkit. Further, as we will argue later, it is not just plants that have such fundamental TRGs. Nearly every clade does. These fundamental TRGs have been largely left out of the current interest in the more narrowly defined TRGs associated with recent novelty, but they should not be. We essentially broaden the current discussion of toolkits and recent TRGs (primarily orphans) to include these fundamental TRGs to better show the central role of TRGs in evolution and biology.

## Studies of TRGs Past and Present

There are now many experimental demonstrations of how important TRGs are to numerous cases of phenotypic novelty (Toll-Riera et al., 2009; Johnson and Tsutsui, 2011; Tautz and Domazet-Loso, 2011; Ding et al., 2012; Ranz and Parsch, 2012; Li et al., 2013; Giarola et al., 2015; Mikheyev and Linksvayer, 2015; Babonis et al., 2016). We do not have space here to do justice to so much work, so we will review three studies that are representative of work on recent and ancient TRGs.

One of the earliest demonstrations of the importance of TRGs was on hydra showing that cnidocytes, specialized stinging cells, are dependent both for their development, and function on TRGs limited to cnidarians (Khalturin et al., 2008, 2009). Essentially, the age-old question is whether novel traits are due to the novel use of conserved genes or the evolution of novel genes and this study showed that both play important roles. Two factors are key here with respect to the current work. First, cnidocytes are lineage specific and of fundamental life-history importance to cnidarians. Second, this case of novelty is ancient in that the cnidarians split from the other Metazoa hundreds of millions of years ago. Hence, lineage specific genes arose in the earliest evolutionary radiations and have been central to their clades ever since.

The second case is associated with gall forming insects. These insects induce plants to produce a domicile, called a gall, in which the insect lives and feeds (Zhao et al., 2015). How the insects induce plants to produce these often-elaborate structures, of no use to the plant, has long been a mystery. A pressing questions is whether the mechanism is simple or complex (based on many genes) and whether the relevant genes are unique to gall producers and have evolved for this purpose? Recently, Zhao et al. (2015) showed that a gall producing insect genome contains thousands of novel secreted proteins. Hence, the production of the gall likely has a complex genetic basis and the relevant genes include many TRGs. The key point with respect to this case is that enormous radiations of lineage specific genes can evolve to facilitate novel phenotypes.

The final case comes from our own work on honey bees and is meant to illustrate the recent cases of novelty and TRGs. Social insects are radically derived relative to their solitary ancestors (Johnson and Tsutsui, 2011). Their castes, elaborate nests, and complex communication systems are largely without precedent in solitary wasps and bees. Jasper et al. (2015) showed that most tissues underlying novel social phenotypes are dependent on high expression of TRGs. These genes fall into many classes including orphans, Hymenoptera-specific, and Insect-specific genes. The production of novel secretions to feed the young, chemical communication, and venom tailored for defense against vertebrates are some of the relevant traits dependent on TRGs.

## Why the Relative Neglect of TRGs in Favor of Toolkit Genes?

**FIGURE 1:**
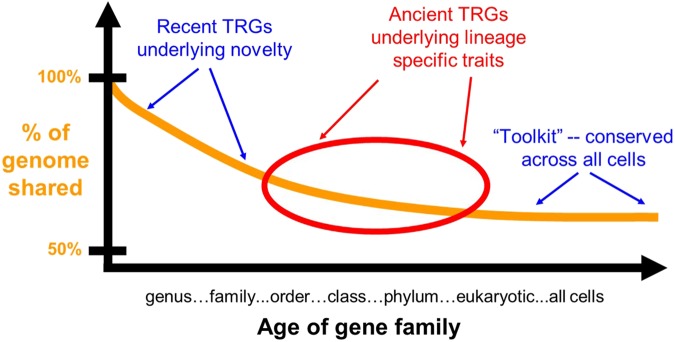
TRGs underlying recent novelty are shared with near taxonomic groups only, while ancient TRGs underlying lineage specific traits are shared by larger groups such as a whole phylum. The toolkit, in the broadest sense, is shared across all organisms. All three classes of genes play major roles in evolution.

Genomes contain genes common to all organisms (the toolkit in the broadest sense), genes limited to some clades only, and genes in just that genome (**Figure 1**). There is some gray area here, however, particularly with respect to how these terms are used in practice. A gene found in all animals, but missing from plants and fungi, for example, could be thought of as a toolkit gene for the animals or a TRG limited to the animals. Such semantics aside, the notion of a toolkit and a TRG remains useful if one is clear about the distinction between them. For our present purposes, when we say TRG we mean a gene not present outside the clade with the trait of interest. A toolkit gene is therefore a gene present outside the lineage with the trait of interest. Our discussion is therefore trait (function) centric, not taxonomically centered.

No matter how one defines toolkit, most studies in biology are on the most conserved genes. We argue this is for two reasons. The first has to do with the impact of translational research on biology and the second on a more slippery notion having to do with what we can call the “same vs. different” problem. With respect to translational research, the point is not to downplay the importance of medical research. Rather, the point is to consider whether our justifiable emphasis on human biology clouds our judgment as to the scope of the importance of biological processes not relevant to human biology? When we study just those parts of the fruit fly’s genome shared with humans, for example, are we exploring the biology of the fly or are we using the fly as a tool for understanding those basic processes the fly shares with human beings? Could there not also be many insect-specific biological mechanisms that are central to fly biology that we omit when we take this approach?

The second reason for the bias toward studies of toolkit genes pertains to the question of what is more important: what is conserved between two species or what is different? It is conjecture, but a reason for the emphasis on what is the same vs. what is different may have to do with the intellectual comfort ones derives from sticking to what we know. Suppose we wish to begin work on some organism in a clade which has received little study. What problems are going to be immediately amenable to study? The answer is that if we stick to what is conserved, then many traits are going to be amenable to study. To study what is unique to this clade, however, requires starting over, as the model system work is largely useless in such cases.

## What Makes Animals Different: Lineage Specific Traits and Genes

According to the toolkit paradigm, not only are basic cellular processes dependent on toolkit genes, but even novel traits are produced by the novel use of the conserved toolkit (Carroll, 1995; Wittkopp et al., 2004; Wagner and Lynch, 2008; Brakefield, 2011). In keeping with this view, if a gene is found in thousands of species in many clades but is missing from many more thousands of other species, then it cannot be a toolkit gene. Genes with such distributions would rather be TRGs of broad conservation, that is, they are common to particular clades and those clades are quite large. Further, what defines TRGs is both their lack of universality, and their clear association with important lineage specific traits.

In this section we will elaborate on this basic idea with some examples. We will review some cases that show that quite a few of the most important genes on earth from any perspective, other than medical science, are TRGs.

### Photosynthesis

**FIGURE 2:**
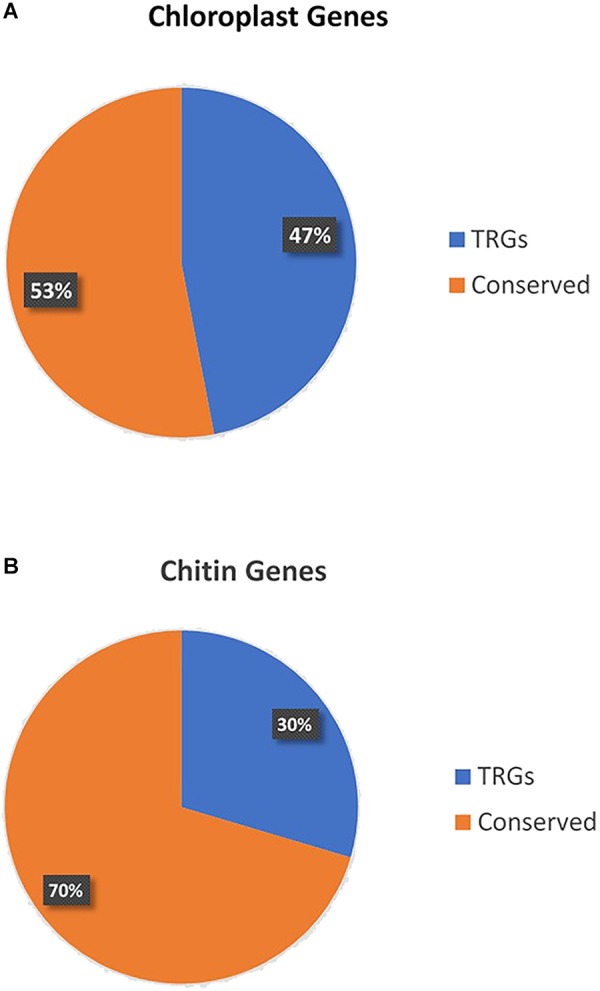
**(A)** Percentage of genes expressed in the chloroplast of *Arabidopsis thaliana* that are not found in non-photosynthesizing metazoans. **(B)** Percentage of genes with GO term ‘chitin metabolism’ that are lineage specific to arthropods or highly conserved across the tree of life.

There can be no more important group of genes than those at the base of nearly all food chains. The pathways that are associated with photosynthesis are complex and many enzymes and other genes with supportive roles are involved (Lambers et al., 2008). There is also great diversity in pathways across the many groups of photosynthetic organisms. However, broad as the conservation of photosynthetic genes are, they are nevertheless TRGs missing from most species across the tree of life. To illustrate this, we downloaded from NCBI all proteins in *Arabidopsis thaliana* with cellular location ‘chloroplast’ and blasted them (*E*-value cutoff 10^-4^) against all Metazoa proteins in the nr NCBI protein database (omitting plants). We used blast (the most common method) to identify homologs, but it should be kept in mind that false negatives are possible with this approach and that similarity of sequence, or homologous status, is not necessarily proof of conservation of function (Studer and Robinson-Rechavi, 2009; Gabaldón and Koonin, 2013). Over half of the 166 proteins (47%) are TRGs, in this case missing from non-photosynthetic, or non-carbon-fixing, organisms (**Figure 2**; genes and taxonomic status in **Supplementary Table S1**). The TRGs include many key genes in photosystems 1 and 2. The most common enzyme on earth, RuBisCo, in fact, is a TRG. In a nutshell, non-photosynthetic organisms do not have photosynthesis genes. While this sounds obvious to the point of being trivial, its repercussions for the question of the general importance of TRGs has never been considered because no one has pointed out how many fundamental biological processes are lineage-specific in nature.

### Structural Support

Organisms make use of a wide variety of compounds to produce support structures to buttress and protect their bodies. TRGs are central to many of these pathways. Cellulose, for example, is the most abundant biological compound on earth. Plants use it for structural support and defense and many animals eat it as the bulk of their diet. Organisms that make use of cellulose have many TRGs associated with its metabolism (Somerville, 2006; Watanabe and Tokuda, 2010). Organisms that do not make use of cellulose lack these genes. Further, the plant cell wall is built of several recalcitrant compounds and the argument for the lineage specificity of the cellulose pathways can be made for the other compounds as well.

Most arthropods use chitin to provide strength to their exoskeletons. Chitin metabolism is based on large pathways of genes with many key actors being TRGs. Chitin metabolism is found across the arthropods, and a few other clades such as fungi, but is missing across most of the tree of life (Bulawa, 1993; Merzendorfer, 2006). We repeated the simple analysis conducted for photosynthesis for this case by downloading all *Drosophila melanogaster* proteins with the GO term ‘chitin metabolism’ from Flybase and then determining what percentage of these 125 proteins are not found in non-arthropod metazoans (blasting again all proteins in the nr NCBI protein database excluding those in the Arthropoda). 29.6% of these important genes are found only in the arthropods (**Figure 2**; genes and taxonomic status in **Supplementary Table S2**).

### Other Cases

There is not space to do justice to the vast array of lineage specific traits that are dependent on TRGs. We could go on to review organisms with internal skeletons like ourselves, or shells of various sorts as in the mollusks. The principles that make insect and plant structural systems based on TRGs are true here as well. We already mentioned the cnidarian stinging cells being key to their clade. Immune systems also vary widely in basic design and function across the tree of life and many key players are TRGs (Sackton et al., 2013; Jasper et al., 2015). These are all ancient TRGs of fundamental importance to large clades, but the host of studies on recent phenotypic novelty and its dependence on TRGs can be added to this list.

To summarize, lineage specific traits define the differences between clades across the tree of life. There are many different ways to make a living and basic life history strategies that define various clades are often based on TRGs. Whether we are discussing photosynthesis or the structural pathways associated with producing various skeletons, many key genes in the relevant genetic pathways are not part of a toolkit of genes. Molting is key to fruit fly biology, but it is not studied by those interested in medicine because the pathways are limited to insects. If it were just the case that insect have some unique but important traits we could ignore this, but it is rather the cases that most lineages have their own unique pathways that are central to their biology.

## Origin and Function of TRGs

A gene may have a simple evolutionary history or one that is long and convoluted. A gene may evolve *de novo* from non-coding sequence for a particular purpose, for example, and retain that purpose. In contrast, a gene could change so completely that all practical notion of homology is lost. Essentially, a duplication could occur and one paralog could take a new function so unrelated to the old function that after considerable time no amino acid similarity remains. In this last case, do we learn anything about the function of the gene from the study of its evolutionary ancestors? This is why Tinbergen’s levels of analysis notion remains useful in biology (Tinbergen, 1963; Sherman, 1988). A trait, or gene’s, origin, elaboration, and currently utility are technically separate questions.

Of course, in practice, it is often the case that genes retain enough of their function for the study of their history to inform us about current function. The point, therefore, is that it is important to keep in mind that the origin and the current utility of a gene may or may not be linked. In the present paper, we have limited our discussion to issues related to function, not evolutionary history. Whether a given TRG arose *de novo* for a linage specific trait (like the gall forming genes), or whether a TRG arose for one function long ago, but evolved a lineage specific function later (like mammalian milk proteins) is not pivotal to the present discussion.

The present discussion focusses on whether lineage specific traits have genetic bases that are common to all organisms or limited to the lineage in which they occur? For this purpose, it is does not matter whether the relevant TRGs have distant homologs with different functions outside the lineage. What matters is whether the genes have homologs with the same function outside the lineage. For the cases discussed here, and many others, it is the case that the TRGs play roles that are unique to the clades that have them and one cannot study these traits or the genes that underlie them in model systems in different clades.

## TRGs in Evolutionary Genetics

The main benefit of an approach that balances the roles of both toolkit genes and TRGs can be illustrated with some discussion of the field of Evo-devo (Carroll, 1995; Wittkopp et al., 2004; Wagner and Lynch, 2008; Brakefield, 2011). Most work in this field seeks to identify the key transcription factors that trigger the production of a novel developmental pattern. It may be the case, for example, that transcription factor (TF) A is expressed at the critical time and place in limb type 1 but TF B is expressed at the same critical point in limb type 2. This approach has been enormously fruitful, but what is often missing is a demonstration of how conserved the rest of the pathway is downstream from that key signal. Novelty is likely at the more distal ends of the gene networks, particularly in secreted proteins (Krylov et al., 2003; Yang et al., 2005; Julenius and Pedersen, 2006; Ramsay et al., 2009; Franzenburg et al., 2013; Jasper et al., 2015). Genes that are lost or radically change at these distal branches do not produce deleterious side effects elsewhere thus freeing up this portion of the network for radical evolutionary change. In short, until we begin to map out complete pathways underlying conserved and lineage specific traits, it is premature to say whether toolkit genes or TRGs are more important for any particular trait.

## Conclusion

Common molecular machinery is conserved across the tree of life and it is easy to understand why its study has dominated research in biology and evolution. However, it is also the case that every major lineage across the tree of life has unique biology limited to its clade and the nature of this limitation is that both the phenotypes and the genes that encode them are lineage specific. Hence, understanding both the conserved and the unique aspects of biological systems are complementary goals for the study of how biological systems evolve and function.

## Author Contributions

BJ conceived and wrote the paper.

## Conflict of Interest Statement

The author declares that the research was conducted in the absence of any commercial or financial relationships that could be construed as a potential conflict of interest.

## Supplementary Material

TABLE S1 *Arabidopsis* genes expressed in the chloroplast and their degree of taxonomic restriction.

TABLE S2 *Drosophila melanogaster* genes with GO ‘chitin metabolism’ and their degree of taxonomic restriction.

## References

[B1] Babonis L. S. Martindale M. Q. Ryan J. F. (2016). Do novel genes drive morphological novelty? An investigation of the nematosomes in the sea anemone *Nematostella vectensis*. *BMC Evol. Biol.* 16:114. 10.1186/s12862-016-0683-3 27216622 PMC4877951

[B2] Begun D. J. Lindfors H. A. Kern A. D. Jones C. D. (2007). Evidence for de novo evolution of testis-expressed genes in the *Drosophila yakuba* *Drosophila erecta* clade. *Genetics* 176 1131–1137. 10.1534/genetics.106.069245 17435230 PMC1894579

[B3] Brakefield P. M. (2011). Evo-devo and accounting for Darwin’s endless forms. *Philos. Trans. R. Soc. Lond. B Biol. Sci.* 366 2069–2075. 10.1098/rstb.2011.0007 21690125 PMC3130370

[B4] Bulawa C. E. (1993). Genetics and molecular biology of chitin synthesis in fungi. *Annu. Rev. Microbiol.* 47 505–534. 10.1146/annurev.mi.47.100193.002445 8257107

[B5] Carroll S. B. (1995). Homeotic genes and the evolution of arthropods and chordates. *Nature* 376 479–485. 10.1038/376479a0 7637779

[B6] Ding Y. Zhou Q. Wang W. (2012). Origins of new genes and evolution of their novel functions. *Annu. Rev. Ecol. Evol. Syst.* 43 345–363. 10.1146/annurev-ecolsys-110411-160513

[B7] Franzenburg S. Walter J. Kunzel S. Wang J. Baines J. F. Bosch T. C. G. (2013). Distinct antimicrobial peptide expression determines host species-specific bacterial associations. *Proc. Natl. Acad. Sci. U.S.A.* 110 E3730–E3738. 10.1073/pnas.1304960110 24003149 PMC3785777

[B8] Gabaldón T. Koonin E. V. (2013). Functional and evolutionary implications of gene orthology. *Nat. Rev. Genet.* 14 360–366. 10.1038/nrg3456 23552219 PMC5877793

[B9] Giarola V. Krey S. Frerichs A. Bartels D. (2015). Taxonomically restricted genes of *Craterostigma plantagineum* are modulated in their expression during dehydration and rehydration. *Planta* 241 193–208. 10.1007/s00425-014-2175-2 25262421

[B10] Jasper W. C. Linksvayer T. A. Atallah J. Friedman D. Chiu J. C. Johnson B. R. (2015). Large-scale coding sequence change underlies the evolution of post developmental novelty in honey bees. *Mol. Biol. Evol.* 32 334–346. 10.1093/molbev/msu292 25351750

[B11] Johnson B. R. Tsutsui N. D. (2011). Taxonomically restricted genes are associated with the evolution of sociality in the honey bee. *BMC Genomics* 12:164. 10.1186/1471-2164-12-164 21447185 PMC3072959

[B12] Julenius K. Pedersen A. G. (2006). Protein evolution is faster outside the cell. *Mol. Biol. Evol.* 23 2039–2048. 10.1093/molbev/msl081 16891379

[B13] Khalturin K. Anton-Erxleben F. Sassmann S. Wittlieb J. Hemmrich G. Bosch T. C. G. (2008). A novel gene family controls species-specific morphological traits in hydra. *PLoS Biol.* 6:e278. 10.1371/journal.pbio.0060278 19018660 PMC2586386

[B14] Khalturin K. Hemmrich G. Fraune S. Augustin R. Bosch T. C. G. (2009). More than just orphans: are taxonomically-restricted genes important in evolution? *Trends Genet.* 25 404–413. 10.1016/j.tig.2009.07.006 19716618

[B15] Krylov D. M. Wolf Y. I. Rogozin I. B. Koonin E. V. (2003). Gene loss, protein sequence divergence, gene dispensability, expression level, and interactivity are correlated in eukaryotic evolution. *Genome Res.* 13 2229–2235. 10.1101/gr.1589103 14525925 PMC403683

[B16] Lambers H. Chapin F. S. Pons T. L. (2008). *Photosynthesis. Plant Physiological Ecology.* New York, NY: Springer Press 10.1007/978-0-387-78341-3

[B17] Li J. W. Lehmann S. Weissbecker B. Naharros I. O. Schutz S. Joop G. (2013). Odoriferous defensive stink gland transcriptome to identify novel genes necessary for quinone synthesis in the red flour beetle, *Tribolium castaneum*. *PLoS Genet.* 9:e1003596. 10.1371/journal.pgen.1003596 23874211 PMC3708791

[B18] Merzendorfer H. (2006). Insect chitin synthases: a review. *J. Comp. Physiol. B* 176 1–15. 10.1007/s00360-005-0005-3 16075270

[B19] Mikheyev A. S. Linksvayer T. A. (2015). Genes associated with ant social behavior show distinct transcriptional and evolutionary patterns. *eLife* 4:e04775. 10.7554/eLife.04775 25621766 PMC4383337

[B20] Ramsay H. Rieseberg L. H. Ritland K. (2009). The correlation of evolutionary rate with pathway position in plant terpenoid biosynthesis. *Mol. Biol. Evol.* 26 1045–1053. 10.1093/molbev/msp021 19188263

[B21] Ranz J. M. Parsch J. (2012). Newly evolved genes: moving from comparative genomics to functional studies in model systems. *Bioessays* 34 477–483. 10.1002/bies.201100177 22461005

[B22] Sackton T. B. Werren J. H. Clark A. G. (2013). Characterizing the infection-induced transcriptome of *Nasonia vitripennis* reveals a preponderance of taxonomically-restricted immune genes. *PloS One* 8:e83984. 10.1371/journal.pone.0083984 24386321 PMC3873987

[B23] Sherman P. W. (1988). The levels of analysis. *Anim. Behav.* 36 616–618. 10.1016/S0003-3472(88)80039-3

[B24] Shigenobu S. Stern D. L. (2013). Aphids evolved novel secreted proteins for symbiosis with bacterial endosymbiont. *Proc. R. Soc. Lond. B* 280:20121952. 10.1098/rspb.2012.1952 23173201 PMC3574423

[B25] Somerville C. (2006). Cellulose synthesis in higher plants. *Annu. Rev. Cell Dev. Biol.* 22 53–78. 10.1146/annurev.cellbio.22.022206.160206 16824006

[B26] Studer R. A. Robinson-Rechavi M. (2009). How confident can we be that orthologs are similar, but paralogs differ? *Trends Genet.* 25 210–216. 10.1016/j.tig.2009.03.004 19368988

[B27] Tautz D. Domazet-Loso T. (2011). The evolutionary origin of orphan genes. *Nat. Rev. Genet.* 12 692–702. 10.1038/nrg3053 21878963

[B28] Tinbergen N. (1963). On aims and methods in ethology. *Z. Tierpsychol.* 20 410–433. 10.1111/j.1439-0310.1963.tb01161.x

[B29] Toll-Riera M. Bosch N. Bellora N. Castelo R. Armengol L. Estivill X. (2009). Origin of primate orphan genes: a comparative genomics approach. *Mol. Biol. Evol.* 26 603–612. 10.1093/molbev/msn281 19064677

[B30] Voolstra C. R. Sunagawa S. Matz M. V. Bayer T. Aranda M. Buschiazzo E. (2011). Rapid evolution of coral proteins responsible for interaction with the environment. *PLoS One* 6:e20392. 10.1371/journal.pone.0020392 21633702 PMC3102110

[B31] von Reumont B. M. Campbell L. I. Richter S. Hering L. Sykes D. Hetmank J. (2014). A polychaete’s powerful punch: venom gland transcriptomics of *Glycera* reveals a complex cocktail of toxin homologs. *Genome Biol. Evol.* 6 2406–2423. 10.1093/gbe/evu190 25193302 PMC4202326

[B32] Wagner G. P. Lynch V. J. (2008). The gene regulatory logic of transcription factor evolution. *Trends Ecol. Evol.* 23 377–385. 10.1016/j.tree.2008.03.006 18501470

[B33] Watanabe H. Tokuda G. (2010). Cellulolytic systems in insects. *Ann. rev. entomol.* 55 609–632. 10.1146/annurev-ento-112408-085319 19754245

[B34] Wilson G. A. Bertrand N. Patel Y. Hughes J. B. Feil E. J. Field D. (2005). Orphans as taxonomically restricted and ecologically important genes. *Microbiology* 151 2499–2501. 10.1099/mic.0.28146-0 16079329

[B35] Wittkopp P. J. Haerum B. K. Clark A. G. (2004). Evolutionary changes in cis and trans gene regulation. *Nature* 430 85–88. 10.1038/nature02698 15229602

[B36] Yang J. Su A. I. Li W. H. (2005). Gene expression evolves faster in narrowly than in broadly expressed mammalian genes. *Mol. Biol. Evol.* 22 2113–2118. 10.1093/molbev/msi206 15987875

[B37] Zhao C. Y. Escalante L. N. Chen H. Benatti T. R. Qu J. X. Chellapilla S. (2015). A massive expansion of effector genes underlies gall-formation in the wheat pest *Mayetiola destructor*. *Curr. Biol.* 25 613–620. 10.1016/j.cub.2014.12.057 25660540

